# Technical-tactical indicators associated with winning in elite amateur boxing: A matched-pair analysis

**DOI:** 10.1371/journal.pone.0359509

**Published:** 2026-09-28

**Authors:** Xuguang Chen, Mohamad Nizam Mohamed Shapie, Wenyao Chen

**Affiliations:** Faculty of Sports Science and Recreation, Universiti Teknologi MARA (UiTM), Shah Alam, Selangor, Malaysia; Sheffield Hallam University, UNITED KINGDOM OF GREAT BRITAIN AND NORTHERN IRELAND

## Abstract

Identifying technical-tactical indicators associated with winning in elite amateur boxing remains challenging because each bout contains a naturally dependent winner-loser pair. Using a matched-pair notational design, we analysed 108 international elite amateur bouts (216 boxer observations) from 2019 to 2025 based on official competition videos and official results. Coded indicators were total punches thrown, punches landed, punching accuracy, successful counterattacks, defensive success rate, ring-centre control and warnings. Winner-loser differences were assessed with paired tests, and independent associations with winning were examined using conditional logistic regression stratified by bout ID, followed by 1000-resample bootstrap internal validation. Compared with losers, winners showed higher punching accuracy (30.41 ± 5.87% vs 26.84 ± 5.79%; mean difference 3.57%, 95% CI 2.10 to 5.05; p < 0.001), more successful counterattacks, higher defensive success rate and higher ring-centre control. In the matched-pair multivariable model, punching accuracy (OR 1.26, 95% CI 1.12 to 1.43), defensive success rate (OR 1.18, 95% CI 1.06 to 1.30), ring-centre control (OR 1.07, 95% CI 1.01 to 1.13) and total punches thrown (OR 1.06, 95% CI 1.02 to 1.09) were independently associated with winning, whereas successful counterattacks were not independently significant after adjustment. The model showed strong internally described discrimination (AUC 0.95, 95% CI 0.91 to 0.98; bootstrap-corrected AUC 0.93) and acceptable prediction error (Brier score 0.09; bootstrap-corrected Brier score 0.11). These findings suggest that winning in elite amateur boxing is associated with an integrated profile combining scoring efficiency, defensive quality, spatial control and sustained offensive output.

## Introduction

Winning in elite amateur boxing emerges from a dynamic interaction of offensive execution, defensive control, spatial positioning and tactical decision-making under time pressure, a demand profile consistent with evidence that task complexity can distinguish expertise in fast, closed-skill sport [1]. Contemporary scoring rewards clear, effective and rule-compliant actions rather than activity volume alone, and related combat-sport work shows that competition responses can vary across preparation status and acute contest demands [2,3]. Although punch volume is often used as a practical performance marker, bout outcomes in modern amateur boxing depend on judged scoring quality, action timing and efficient management of exchanges; related work shows that structural and technical constraints shape technical-tactical profiles rather than raw output alone [4].

Recent notational studies have improved understanding of winner-loser differences in activity profiles, technical effectiveness and sex-specific performance characteristics in amateur boxing [2,5–10]. Related work across combat and fast-response sports also highlights the relevance of perceptual-cognitive skill, transition timing and defensive execution for competitive success [1,11–16]. However, evidence remains mixed regarding the relative importance of offensive volume versus efficiency-oriented indicators when multiple tactical dimensions are considered simultaneously, a tension consistent with high-frequency performance analysis and evidence that technical constraints can alter performance profiles independently of gross output [4,17].

A core methodological issue is that boxing data are naturally paired: each bout contributes one winner and one loser, and these observations are not independent. Match analysis that retains within-contest temporal and participant structure provides a precedent for treating contest observations as structurally linked [18]. Analyses that ignore this dependency may produce biased uncertainty estimates and potentially unstable inference [19,20]. A second gap is that many studies emphasise univariate contrasts, while fewer have jointly modelled offensive efficiency, counterattacking, defensive quality and spatial control in a single matched analytical framework; multidimensional profiling in high-level combat-sport populations illustrates the value of considering interacting performance domains [21].

To address these gaps, this study applied a matched-pair notational framework and conditional logistic regression stratified by bout ID to identify technical-tactical indicators associated with winning in elite amateur boxing. We analysed international elite bouts from 2019 to 2025 and integrated indicators of offensive quantity, offensive efficiency, counterattack success, defensive success and ring-centre control. We expected winners to show stronger efficiency-oriented and control-oriented profiles than losers, and we further examined which indicators remained independently associated with winning after accounting for the within-bout dependency structure.

## Materials and methods

### Study design

This retrospective matched-pair observational study was based on official competition videos and official bout results. The manuscript was prepared with reference to the STROBE reporting guidance for observational studies [22,23]. Because model discrimination and calibration summaries were reported, relevant reporting concepts from TRIPOD and TRIPOD+AI were also considered, while recognizing that the present model was explanatory and internally validated rather than intended as an externally deployable prediction model [24,25].

### Sample and bout selection

An initial sample of 124 elite amateur boxing bouts contested between 2019 and 2025 was screened. Eligible competitions included International Boxing Association men’s and women’s world championships, Asian Boxing Championships, major Olympic boxing tournaments, Commonwealth Games boxing tournaments, Pan American boxing tournaments and European championship-level amateur tournaments. Competition videos were obtained from official competition channels and recognised official replay platforms, including the IBA official YouTube channel, Asian Boxing Confederation official platform, Olympic/NBC replay pages, Commonwealth Games result platforms, European Boxing Confederation pages and Pan American Games result platforms. Bout outcomes were cross-checked against official International Boxing Association records and official competition result books. The video source, official result source and timestamp interval for each bout are provided in S2 File.

Bouts were excluded when video footage was incomplete (n = 8), when the bout ended by withdrawal or retirement and the result was not suitable for analysis (n = 5), or when camera obstruction compromised the coding of key technical-tactical indicators (n = 3). Withdrawal- or retirement-based results were excluded because bout duration and tactical exposure can be truncated by injury, medical stoppage or non-performance factors, making the winner-loser indicator profile less comparable with a completed judged contest. The final analytical sample comprised 108 bouts, corresponding to 108 matched winner-loser pairs and 216 boxer observations.

Inclusion criteria were: (1) official international elite amateur boxing bouts within the specified competitions; (2) clearly documented results with no disputed reversal; (3) complete video footage with no loss of key visual information; and (4) verifiable information on sex, weight category and competition stage. Exclusion criteria were: (1) loss of one or more complete rounds or insufficient viewing angle for the core indicators; (2) bouts terminated by withdrawal, retirement, disqualification or official reversal; and (3) inability to verify bout stage, weight category or boxer information. Following the participant-classification framework proposed by McKay et al. [26], the sample was classified by competition level rather than individual training status; all included bouts were contested at international championship, Olympic, continental or multi-sport-games level. Sex, competition stage and weight category were verified from official result books and event listings. Athlete height and reach were not consistently available in official sources and were therefore not used as covariates.

### Technical-tactical indicators

The coded variables were total punches thrown, punches landed, punching accuracy, successful counterattacks, defensive success rate, ring-centre control and warnings. Punching accuracy was calculated as punches landed divided by total punches thrown multiplied by 100. A successful counterattack was defined as a valid scoring action completed immediately after the opponent’s offensive action and before the exchange reset. Defensive success rate was defined as the proportion of successful blocks, parries, evasions, retreats or other defensive responses among all observed defensive responses. Ring-centre control was defined as the proportion of observable bout time in which a boxer occupied and tactically controlled the central ring area while directing the exchange or limiting the opponent’s preferred movement route. For coding purposes, the centre was treated as the central tactical zone of the visible ring; control required sustained occupation and initiative for at least a continuous two-second segment rather than a momentary step through the centre. Warnings were coded as official referee warnings recorded during the bout.

The coding manual was developed from standardised frameworks used in boxing and sport performance analysis [27,28] and from previous elite boxing studies [2,6]. It specified inclusion and exclusion criteria, operational boundaries for each indicator, coding workflows and representative decision examples. The expanded coding manual and operational definitions are provided as S1 File, and the anonymised coded dataset is provided as S1 Dataset.

### Coding procedure and reliability

Two analysts with boxing-specific backgrounds independently coded all bouts according to the standardised coding manual. To reduce observational bias, coders were not informed of the official bout outcomes during technical-tactical coding. Outcome labels were assigned by the research team only after technical-tactical coding had been completed and checked against official results. Video review was performed through the official replay platforms listed in S2 File, with timestamps recorded to the nearest second in spreadsheet templates. The heterogeneous third-party streams did not share a single acquisition frame rate, and exact frame-rate metadata were not retained in the coding archive. Coding therefore used elapsed playback time at normal speed rather than frame counts; the two-second ring-centre-control rule was applied using the platform time display. No automated computer-vision tracking, motion-capture system or proprietary kinematic analysis package was used.

Before formal coding, three pilot-coding sessions were conducted to standardise operational criteria. Disagreements between coders were reviewed after independent coding and resolved through consensus discussion; unresolved decisions were reviewed by a researcher with boxing-specific expertise. To assess reliability, 20 bouts (18.5% of the sample) were randomly selected for repeat coding after a 10-day interval. The same analysts performed the repeat coding using the same operational definitions. Reliability coefficients are reported as measurement-model results in the Results section.

### Statistical analysis

Statistical analyses were performed in R using the survival package for conditional logistic regression and custom rank-based functions in S1 Code for discrimination summaries. The complete R workflow is provided as S1 Code and writes R session information to support reproducibility. No missing values were present in the final analytical dataset. Data are reported as mean and SD unless otherwise stated. Statistical significance was set at alpha = 0.05. Because each bout included one winner and one loser, univariate comparisons used paired statistical methods. Paired differences were inspected using distributional plots and normality diagnostics before test selection. Paired-sample t-tests were used for approximately normally distributed paired differences, whereas Wilcoxon signed-rank tests were used for non-normal or discrete paired differences. Effect sizes were reported as Cohen’s dz for t-tests and r for Wilcoxon signed-rank tests [29,30]. Because six planned paired contrasts were examined, exact p-values are reported and a Benjamini-Hochberg false-discovery-rate sensitivity check was also conducted; this did not change the interpretation of which paired contrasts were statistically significant.

Multivariable analysis used conditional logistic regression stratified by bout ID [19,20]. Candidate variables included punching accuracy, successful counterattacks, defensive success rate, ring-centre control and total punches thrown. Punches landed were excluded from the main model because of potential collinearity with total punches thrown and punching accuracy. Model results are reported as beta coefficients, standard errors, odds ratios, 95% confidence intervals and p-values, with confidence intervals interpreted as estimates of precision rather than dichotomous tests alone [31,32]. Within-sample model performance was summarised using the area under the receiver operating characteristic curve, Brier score, calibration intercept and calibration slope [33,34]. Because a conditional logistic model stratified by bout has no shared marginal intercept, discrimination and calibration were calculated from a pair-symmetric score based on winner-minus-loser covariate differences and the fitted coefficients. These performance summaries should therefore be interpreted as internal summaries of the paired model, not as externally validated individual-level probabilities. Internal validation was performed using 1000 bootstrap resamples at the matched-pair level. For each bootstrap sample, the conditional logistic model was refitted and apparent-versus-test optimism was estimated for AUC and Brier score; optimism-corrected values are reported.

No post-hoc power analysis was used to reinterpret statistically non-significant findings. As a planning-scale sensitivity statement, 108 paired bouts provide approximately 80% power at two-sided alpha = 0.05 to detect a paired standardised mean difference of about dz = 0.27. Subgroup analyses were exploratory, and formal interaction tests were not conducted for all subgroup comparisons; therefore, subgroup findings were not interpreted as definitive stratified conclusions.

### Ethics statement

This study used publicly available official competition videos and official results, involved no direct contact with athletes and collected no non-public personal information. Therefore, it was considered a non-interventional analysis of publicly available data, and additional institutional ethics approval and participant consent were not required.

## Results

### Sample characteristics

The final sample included 108 elite amateur boxing bouts and 216 boxer observations. There were 124 male observations (57.4%) and 92 female observations (42.6%). By weight category, 78 observations were from lightweight divisions (36.1%), 72 from middleweight divisions (33.3%) and 66 from heavyweight divisions (30.6%). By competition stage, 78 observations were from preliminary bouts (36.1%), 60 from quarter-finals (27.8%), 44 from semi-finals (20.4%) and 34 from finals (15.7%) (Table 1).

**Table 1:** Sample characteristics.

Characteristic	n	%
Male observations	124	57.4
Female observations	92	42.6
Lightweight divisions	78	36.1
Middleweight divisions	72	33.3
Heavyweight divisions	66	30.6
Preliminary bouts	78	36.1
Quarter-finals	60	27.8
Semi-finals	44	20.4
Finals	34	15.7

### Reliability results

Repeat coding of 20 bouts (18.5% of the sample) produced intraclass correlation coefficients of 0.91 for punches landed, 0.89 for punching accuracy and 0.93 for ring-centre control, together with Cohen’s kappa of 0.86 for warnings (Table 2). These own-sample repeat-coding results support the reproducibility of the retained measurement procedures and follow established reliability-reporting principles in sport measurement [35,36]. Separate archived recoding matrices were not available for successful counterattacks or defensive success rate; no retrospective or simulated coefficients were substituted for those missing empirical results.

**Table 2:** Repeat-coding reliability results.

Indicator	Repeat-coding result
Punches landed	ICC = 0.91
Punching accuracy	ICC = 0.89
Ring-centre control	ICC = 0.93
Warnings	Cohen’s kappa = 0.86
Successful counterattacks	Archived recoding matrix unavailable; coefficient not estimated
Defensive success rate	Archived recoding matrix unavailable; coefficient not estimated

Note. Twenty bouts (18.5% of the sample) were recoded after a 10-day interval. ICC = intraclass correlation coefficient.

### Paired comparisons between winners and losers

Paired comparisons showed that winners outperformed losers across several technical-tactical indicators (Table 3; Figs 1 and 2). Winners showed significantly higher punching accuracy (30.41 ± 5.87% vs 26.84 ± 5.79%; mean difference = 3.57%, 95% CI 2.10 to 5.05; t = 4.81, p < 0.001, dz = 0.46). Winners also showed more successful counterattacks (5.47 ± 1.72 vs 4.24 ± 1.56; mean difference = 1.23; Z = −4.85, p < 0.001, r = 0.52), higher defensive success rate (61.87 ± 5.99% vs 57.10 ± 5.20%; mean difference = 4.77%, 95% CI 3.22 to 6.33; t = 6.08, p < 0.001, dz = 0.59) and higher ring-centre control (56.69 ± 8.36% vs 50.96 ± 9.15%; mean difference = 5.73%, 95% CI 3.55 to 7.91; t = 5.21, p < 0.001, dz = 0.50). Total punches thrown showed a small, non-significant paired difference (151.92 ± 21.28 vs 146.69 ± 23.12; mean difference = 5.22, 95% CI −0.35 to 10.79; t = 1.86, p = 0.066, dz = 0.18). The Benjamini-Hochberg sensitivity check did not change the interpretation: all paired contrasts except total punches thrown remained statistically significant.

**Table 3:** Paired comparison of technical-tactical indicators between winners and losers.

Variable	Winners, mean ± SD	Losers, mean ± SD	Mean difference	95% CI	Test statistic	p-value	Effect size
Total punches thrown	151.92 ± 21.28	146.69 ± 23.12	5.22	−0.35 to 10.79	t = 1.86, df = 107	0.066	dz = 0.18
Punches landed	45.27 ± 5.70	38.56 ± 6.42	6.71	5.12 to 8.31	t = 8.35, df = 107	<0.001	dz = 0.80
Punching accuracy (%)	30.41 ± 5.87	26.84 ± 5.79	3.57	2.10 to 5.05	t = 4.81, df = 107	<0.001	dz = 0.46
Successful counterattacks	5.47 ± 1.72	4.24 ± 1.56	1.23	–	Z = −4.85	<0.001	r = 0.52
Defensive success rate (%)	61.87 ± 5.99	57.10 ± 5.20	4.77	3.22 to 6.33	t = 6.08, df = 107	<0.001	dz = 0.59
Ring-centre control (%)	56.69 ± 8.36	50.96 ± 9.15	5.73	3.55 to 7.91	t = 5.21, df = 107	<0.001	dz = 0.50

Note. CI = confidence interval; dz = Cohen’s dz; r = effect size for the Wilcoxon signed-rank test.

**Fig 1:**
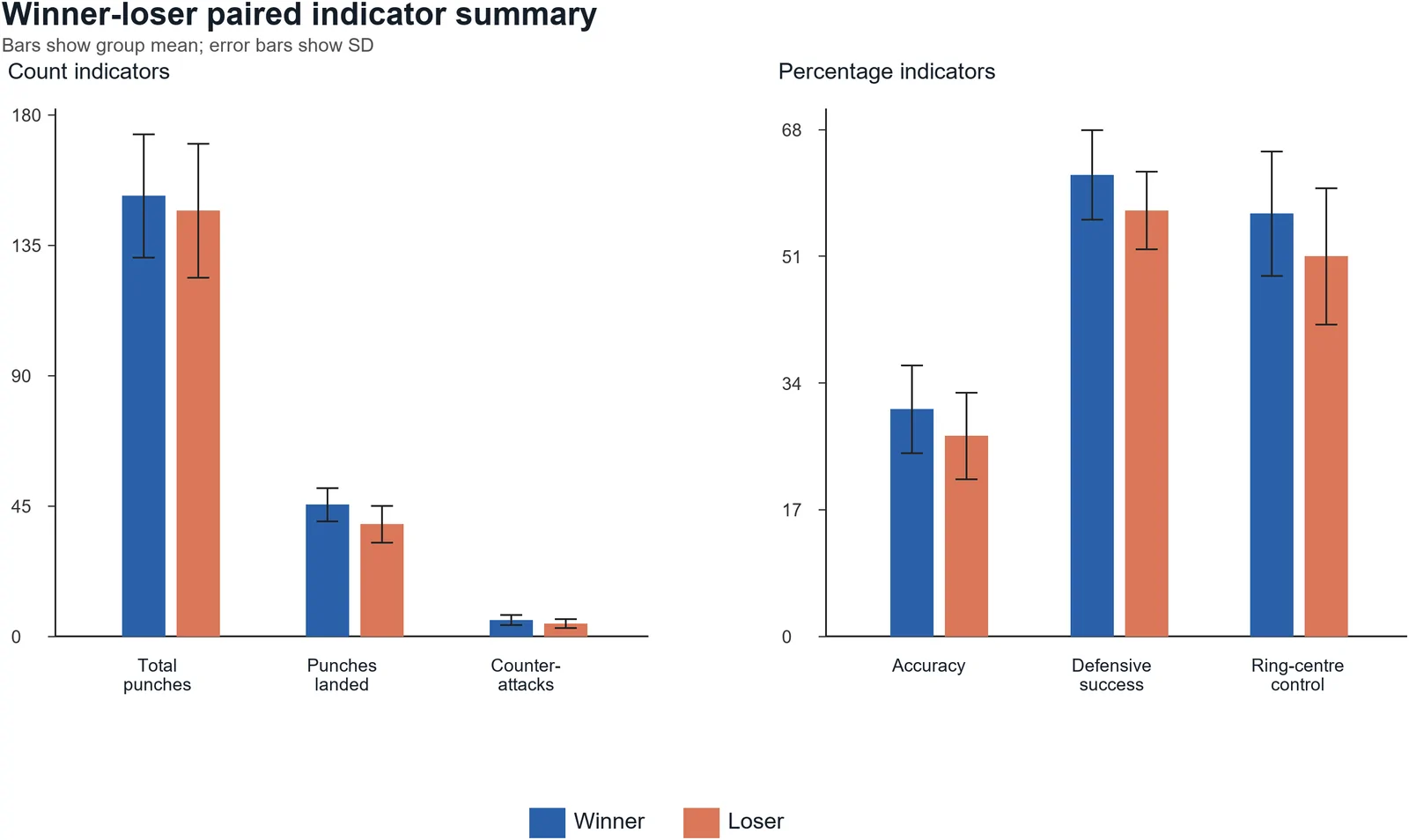
Paired summary of technical-tactical indicators for winners and losers. Bars show group means and error bars show SD. Count-based indicators and percentage-based indicators are shown in separate panels to avoid combining unlike scales.

**Fig 2:**
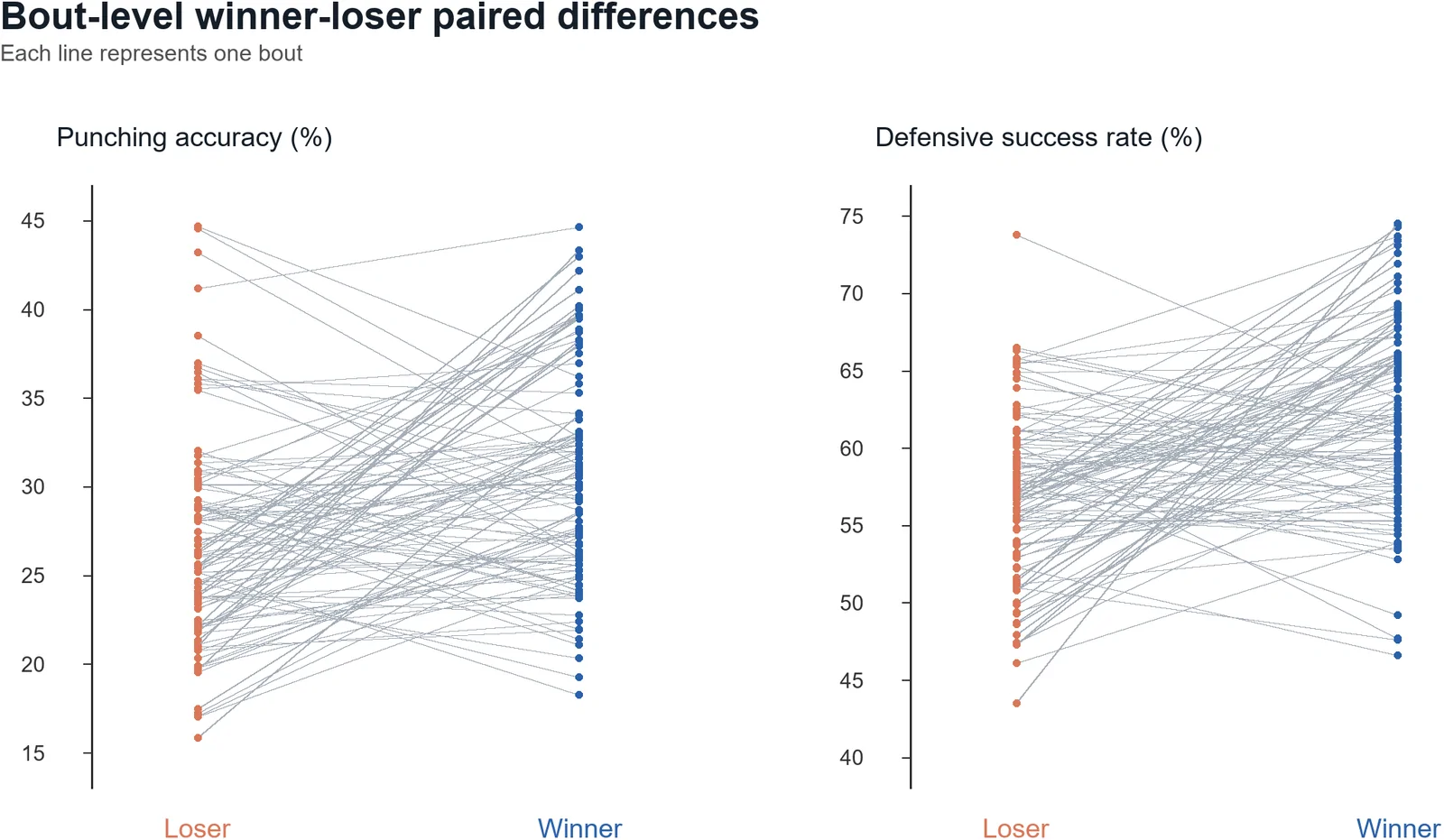
Bout-level paired differences in punching accuracy and defensive success rate. Each line connects the loser and winner from the same bout, preserving the within-bout linkage that underlies the matched-pair analysis.

### Multivariable matched-pair model

The conditional logistic regression model showed that punching accuracy, defensive success rate, ring-centre control and total punches thrown were independently associated with winning (Table 4; Fig 3). Successful counterattacks were positively associated in paired comparisons but were not independently significant after adjustment for the other indicators. Each one-percentage-point increase in punching accuracy was associated with a 26% increase in the odds of winning (odds ratio = 1.26, 95% CI 1.12 to 1.43, p < 0.001). Each one-percentage-point increase in defensive success rate was associated with an 18% increase in the odds of winning (odds ratio = 1.18, 95% CI 1.06 to 1.30, p = 0.002). Ring-centre control remained independently associated with winning (odds ratio = 1.07, 95% CI 1.01 to 1.13, p = 0.020), and total punches thrown showed a modest independent association (odds ratio = 1.06, 95% CI 1.02 to 1.09, p < 0.001). The model demonstrated strong internally described discrimination (area under the curve = 0.95, 95% CI 0.91 to 0.98) and acceptable prediction error (Brier score = 0.09). Bootstrap internal validation (1000 resamples) indicated limited optimism (corrected area under the curve = 0.93; corrected Brier score = 0.11).

**Table 4:** Technical-tactical indicators associated with winning in the multivariable matched-pair model.

Predictor	Beta	SE	Odds ratio	95% CI	p-value
Accuracy	0.2335	0.0622	1.26	1.12 to 1.43	<0.001
Defensive success	0.1620	0.0520	1.18	1.06 to 1.30	0.002
Counterattacks	0.2037	0.1329	1.23	0.94 to 1.59	0.126
Ring-centre control	0.0644	0.0277	1.07	1.01 to 1.13	0.020
Total punches	0.0552	0.0161	1.06	1.02 to 1.09	<0.001

Note. Odds ratios were estimated using conditional logistic regression stratified by bout ID. CI = confidence interval; SE = standard error.

**Fig 3:**
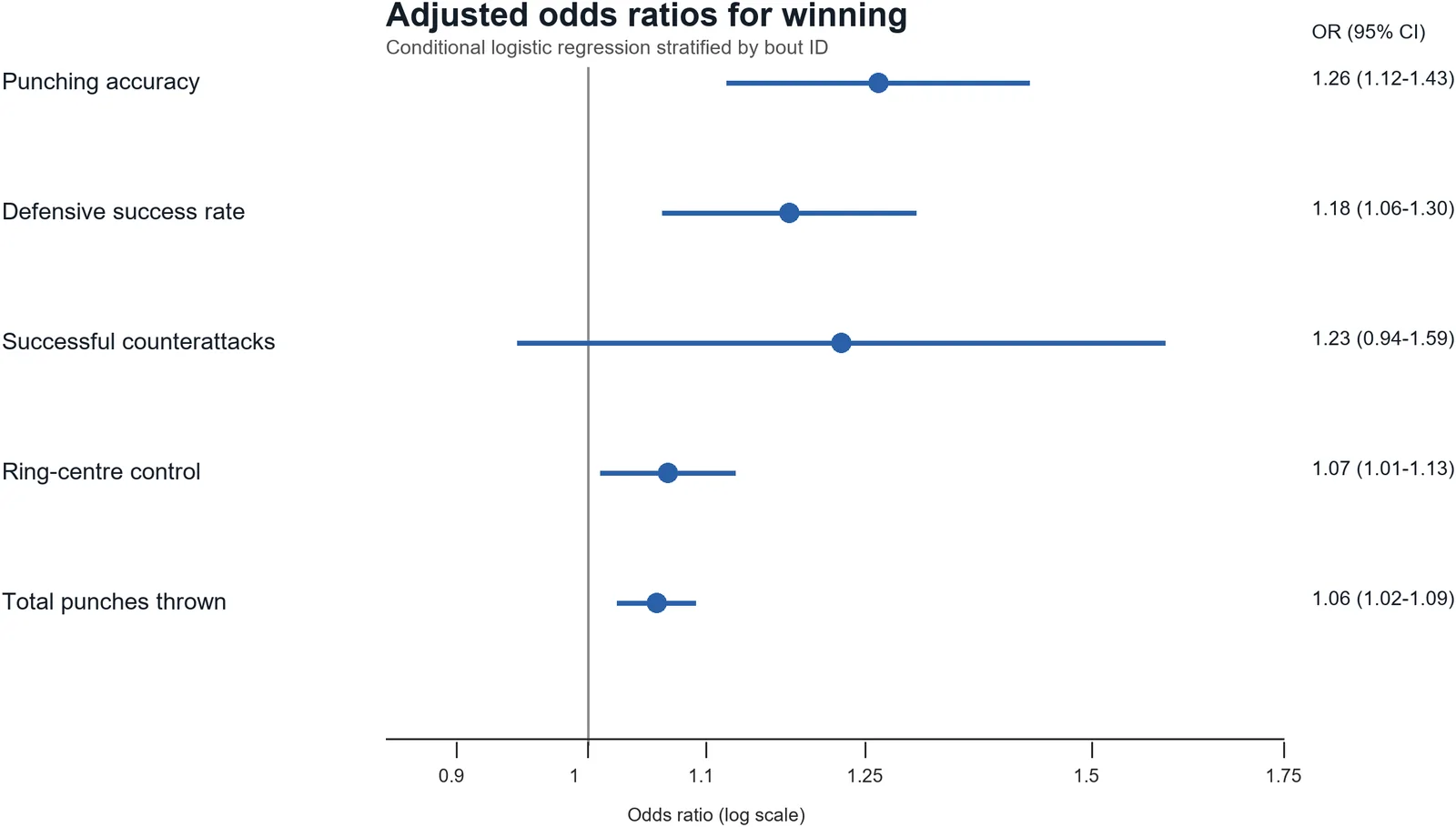
Adjusted odds ratios for winning in the multivariable matched-pair model. Points show odds ratios and horizontal bars show 95% confidence intervals from conditional logistic regression stratified by bout ID. The vertical reference line indicates an odds ratio of 1.

### Exploratory subgroup analysis

Exploratory subgroup conditional logistic analyses suggested that the association patterns between technical-tactical indicators and winning may vary by sex, weight category and competition stage. Because subgroup sample sizes were limited and formal interaction tests were not completed across all subgroup contrasts, these findings should be interpreted as exploratory. The main manuscript therefore emphasises the full-sample matched-pair model and reports subgroup trends as hypothesis-generating evidence for future work. The three-category weight grouping was a pragmatic competition-level summary; alternative anthropometrically grounded classification models may better capture weight-related heterogeneity when height, reach and body-composition data are available [37].

## Discussion

This matched-pair notational study analysed technical-tactical indicators in elite amateur boxing. The main finding was that winning was associated with technical-tactical efficiency and sustained scoring pressure rather than with isolated single indicators. Winners showed higher punching accuracy, successful counterattacks, defensive success rate and ring-centre control in paired comparisons. In the multivariable matched-pair model, punching accuracy and defensive success remained independently associated with winning, while ring-centre control and total punches thrown also contributed after adjustment. These findings indicate that both action quality and the ability to maintain effective offensive output are relevant for explaining bout outcomes at elite level, without implying causal priority among the indicators.

Punching accuracy was the clearest indicator associated with winning. Contemporary amateur boxing scoring emphasises clear, effective and rule-compliant scoring actions. Accordingly, high-frequency but low-efficiency attacking does not necessarily produce a competitive advantage. Dunn et al. reported that winners under the Ten Point Must System showed higher proportions of punches landed, whereas total punching activity or general attacking intent was not necessarily decisive [2]. The present findings are consistent with this view and extend it by showing that punching accuracy remained independently associated with winning in a matched-pair model.

Successful counterattacking showed a clear winner-loser difference, highlighting the tactical value of offensive-defensive transitions. However, its independent effect was attenuated after adjustment for accuracy, defence, spatial control and punch volume, suggesting overlap between transition quality and broader technical efficiency constructs. In this manuscript, transition quality refers to the effectiveness with which a boxer converts the opponent’s attacking action into an immediate scoring opportunity, including timing, defensive completion and counterattack execution.

Defensive success rate contributed independently to winning, indicating that successful boxers not only create scoring opportunities but also reduce the opponent’s ability to score. Defensive quality should be considered an active tactical capability rather than passive avoidance. Together with the univariate advantage in ring-centre control, the findings suggest that successful boxers may maintain initiative by combining defensive stability with spatial positioning, thereby reducing forced retreats and creating better conditions for subsequent counterattacks.

The paired difference in total punches thrown was modest and not statistically significant, but total punches remained independently associated with winning in the adjusted matched-pair model. This pattern should be interpreted cautiously. It suggests that offensive volume may have value when considered alongside scoring efficiency, defensive quality and spatial control, but it does not support treating punch volume as a stand-alone determinant of success.

### Methodological implications for combat-sport notational analysis

A methodological contribution of this study is the use of a matched-pair analytical framework that reflects the dependency structure of boxing bouts. Treating winners and losers from the same bout as independent observations may give misleading estimates of uncertainty. By stratifying analyses by bout, this study compared each winner directly with the corresponding opponent. This approach may be useful for future notational studies in combat sports where each contest naturally creates paired outcome data. The explicit reporting of discrimination, Brier score, calibration and bootstrap optimism also aligns the analysis more closely with transparent model-reporting recommendations [24,25,33].

### Practical implications

The findings offer practical monitoring hypotheses rather than prescriptive training rules. Coaches may consider tracking punching accuracy, effective scoring conversion and defensive success alongside punch volume, because these indicators jointly described winning profiles in this sample. Counterattacking after defensive actions may also be useful to monitor in post-bout review, especially when interpreted together with broader efficiency and spatial-control indicators. Subgroup observations should be used cautiously: they are hypothesis-generating and require validation in larger samples before sex-, weight- or stage-specific training recommendations are made.

### Strengths and limitations

This study has several strengths. First, the data were derived from real international elite competition footage, supporting ecological validity. Second, the matched-pair design aligned the statistical analysis with the natural winner-loser structure of boxing bouts. Third, the study integrated offensive efficiency, counterattacking, defensive performance and spatial control within the same modelling framework. Fourth, the model reported discrimination, calibration and bootstrap internal validation, improving transparency and reproducibility. Fifth, the study deposits the anonymised dataset, analysis code, coding definitions and video-source index as Supporting Information, allowing independent checking of the reported analyses.

Several limitations should be acknowledged. First, the study was based on retrospective video coding and may be influenced by camera angles, broadcast quality and observational judgement, although blind coding procedures and reliability testing were used to reduce bias. Second, the heterogeneous official streams did not share a single acquisition frame rate, and exact frame-rate metadata were not retained in the coding archive; temporal decisions were based on elapsed platform time rather than frame counts. Third, ring-centre control and some defensive indicators involve judgement-based coding. Future studies could incorporate semi-automated computer-vision or instrumented monitoring to improve objectivity, while recognising the methodological and reporting challenges of artificial intelligence and technology-assisted tracking in sport [38–40]. Fourth, separate archived reliability matrices were not available for successful counterattacks and defensive success rate, so separate reliability coefficients for those two model predictors could not be reported; no simulated data were used as empirical reliability evidence. Fifth, potential confounders such as opponent quality, judge tendencies, round-to-round fatigue, injury status, psychological state, height, reach and pre-bout tactical plans were not included. Sixth, subgroup analyses were exploratory and should be validated in larger samples across different continental and competition levels. Seventh, the model underwent internal validation only; external validation in independent competition samples is required before generalising its performance. Finally, as an observational study, the findings should not be interpreted as evidence of causal effects.

## Conclusion

Winning in elite amateur boxing was associated with an integrated technical-tactical profile that combined scoring efficiency, defensive quality, spatial control and sustained offensive output. Punching accuracy and defensive success rate were the most stable independent indicators across analyses, while ring-centre control and total punches thrown also contributed in the adjusted matched-pair model. Successful counterattacks differentiated winners and losers in paired comparisons but did not remain independently significant after adjustment. These findings support matched-pair analytical approaches in boxing notational analysis and suggest that future preparation and monitoring studies should evaluate precision scoring, defensive stability, spatial control and offensive continuity as interacting components rather than isolated determinants.

## Supporting information

S1 Dataset Anonymised coded dataset for the 108 elite amateur boxing bouts and 216 boxer observations. (CSV)

S1 Code R analysis code used for paired tests, false-discovery-rate sensitivity checking, conditional logistic regression, calibration and bootstrap internal validation. (R)

S1 File Coding manual and operational definitions for the technical-tactical indicators. (DOCX)

S2 File Video-source index, official result sources and coding timestamp intervals for the publicly available official competition videos. (XLSX)
