# Supplementary material for: Technical-tactical indicators associated with winning in elite amateur boxing: A matched-pair analysis
Source: PLoS One. 2026 Sep 28;21(9):e0359509. doi: 10.1371/journal.pone.0359509 (PMC13618943; doi:10.1371/journal.pone.0359509)
Supplement: Supplementary file 1 [file pone.0359509.s003.docx]

**S1 File. Coding manual and operational definitions**

Manuscript: Technical-tactical indicators associated with winning in elite amateur boxing: a matched-pair analysis

This file summarises the coding rules used for the deposited anonymised dataset (S1 Dataset) and the video-source index (S2 File).

# Coding workflow

Official bout footage and official result sources were identified before coding. The source platform, URL, official result source and timestamp range for each bout are listed in S2 File.

Two boxing-experienced analysts independently coded the technical-tactical indicators while blinded to the official outcome labels. Winner-loser labels were added only after technical-tactical coding had been completed and checked against official result books.

Disagreements were resolved by consensus. Unresolved cases were reviewed by a researcher with boxing-specific expertise.

Timestamps were recorded to the nearest second. The official streams came from heterogeneous third-party platforms and did not share a single acquisition frame rate; exact frame-rate metadata were not retained in the coding archive. Temporal decisions therefore used elapsed platform time at normal playback speed rather than frame counts, including the continuous two-second ring-centre-control threshold.

Coding used manual notational review of official broadcast footage; no automated tracking or motion-capture system was used.

Count variables in S1 Dataset are reported as whole-number counts. Punching accuracy was recalculated from the deposited integer counts as punches_landed divided by total_punches multiplied by 100.

# Operational definitions

| **Variable** | **Definition** | **Coding rule** |
| --- | --- | --- |
| total_punches | Total punches thrown during the bout. | All observable scoring attempts or clear punch attempts, regardless of whether they landed. |
| punches_landed | Total valid scoring punches landed. | Punches judged as landing clearly on the legal target area based on available broadcast footage. |
| accuracy | Punching accuracy percentage. | Computed from the deposited integer counts as punches_landed / total_punches x 100. |
| counterattacks | Successful counterattacks. | Valid scoring action completed immediately after the opponent's offensive action and before the exchange reset. |
| defensive_success_rate | Successful defensive responses as a percentage of all defensive responses. | Successful block, parry, evasion, retreat or other defensive action preventing or reducing the opponent's scoring opportunity. |
| ring_centre_control | Proportion of observable bout time in which the boxer occupied and controlled the central tactical ring area. | Coded when the boxer sustained central occupation and initiative for at least a continuous two-second segment. |
| warnings | Official warnings. | Referee warnings recorded during the bout. |

# Ring-centre control rule

The central tactical area was treated as the central zone of the visible ring rather than the ropes or corners. A boxer was coded as controlling the centre when they occupied this zone and directed the exchange, pressured the opponent, or limited the opponent's preferred movement route for at least a continuous two-second segment. Momentary steps across the centre without tactical initiative were not coded as centre control.

# Reliability summary

| **Indicator** | **Reliability statistic** |
| --- | --- |
| Punches landed | ICC = 0.91 |
| Punching accuracy | ICC = 0.89 |
| Ring-centre control | ICC = 0.93 |
| Warnings | Cohen's kappa = 0.86 |
| Successful counterattacks | Separate archived recoding matrix not available; no unverified coefficient reported. |
| Defensive success rate | Separate archived recoding matrix not available; no unverified coefficient reported. |

# Source-platform summary

| **Source platform** | **Bouts** |
| --- | --- |
| Asian Boxing Confederation official platform | 36 |
| IBA official YouTube channel | 27 |
| Official Olympic/NBC replay platform | 19 |
| Commonwealth Games official results platform | 12 |
| European Boxing Confederation official platform | 8 |
| Pan American Games official results platform | 6 |
